# E-cadherin, actin, microtubules and FAK dominate different spheroid formation phases and important elements of tissue integrity

**DOI:** 10.1242/bio.037051

**Published:** 2018-12-21

**Authors:** I. Smyrek, B. Mathew, S. C. Fischer, S. M. Lissek, S. Becker, E. H. K. Stelzer

**Affiliations:** Physical Biology/Physikalische Biologie (IZN, FB 15), Buchmann Institute for Molecular Life Sciences (BMLS), Cluster of Excellence Frankfurt – Macromolecular Complexes (CEF – MC), Goethe Universität – Frankfurt am Main (Campus Riedberg), Max-von-Laue-Straße 15, D-60348 Frankfurt am Main, Germany

**Keywords:** Spheroids, Three-dimensional cell culture, Adhesion, Tissue integrity

## Abstract

Spheroids resemble features of tissues and serve as model systems to study cell–cell and cell–ECM interactions in non-adhesive three-dimensional environments. Although it is generally accepted that mature spheroids resemble tissue properties very well, no studies relate different phases in the spheroid formation processes that contribute to tissue integrity. Tissue integrity involves the cellular processes adhesion formation, adhesion reinforcement, rearrangement as well as proliferation. They maintain the structure and function of tissues and, upon dysregulation, contribute to malignancy. We investigated spheroid formation dynamics in cell lines of different metastatic potential. We dissected spheroid formation into phases of aggregation, compaction and growth to identify the respective contributions of E-cadherin, actin, microtubules and FAK. E-cadherin, actin and microtubules drive the first two phases. Microtubules and FAK are involved in the proliferation phase. FAK activity correlates with the metastatic potential of the cells. A robust computational model based on a very large number of experiments reveals the temporal resolution of cell adhesion. Our results provide novel hypotheses to unveil the general mechanisms that contribute to tissue integrity.

## Abstract (precis)

**Summary:** The phases of spheroid formation resemble different stages of cell contact formation. This facilitates studying the temporal contribution of molecules in this process.

## INTRODUCTION

Within the physiological context of tissues, cells behave dynamically in three dimensions. They require an unknown degree of flexibility and robustness to undergo developmental processes like adaption of mammary tissue during pubertal proliferation, or post-lactational regression (Watson and Khaled, 2008). Dysfunctionality can result in cancer (Gray et al., 2010). Hence, tight regulations of cell–cell and cell–ECM interactions, cell migration and cell proliferation are important (Ewald et al., 2008; Gumbiner, 1996). Adhesion formation and maturation and their underlying molecular mechanisms have been studied extensively in cells grown as monolayers, i.e. on flat and rigid surfaces. Such culture conditions influence protein expression, cell morphology and associated processes (Pampaloni et al., 2007). Consequently, tissue-related functions must be studied in non-adhesive environments. During the past two decades, multicellular aggregates, e.g. spheroids, have become the three-dimensional model systems. Spheroids are generated from various cell types and cultured over various periods of time. The process of spheroid formation seems to consist of at least three phases: (1) an initial aggregation of isolated cells is followed by (2) spheroid compaction and (3), finally, spheroid growth (Lin et al., 2006; Enmon et al., 2001).

The involvement of cadherins (Takeichi, 1991) or integrins (Miranti and Brugge, 2002) that connect a cell with its environment has been the main focus in the context of spheroid formation (Lin et al., 2006; Ivascu and Kubbies, 2007; Saias et al., 2015; Enmon et al., 2002; Casey et al., 2001). Only a few studies have investigated the contributions of intracellular components, such as actin or microtubules, to the formation process of three-dimensional cellular aggregates (Tzanakakis et al., 2001; Yoshii et al., 2011). Whereas their involvement in adhesion processes in two-dimensional cultures and in cells cultured on matrix-coats is well-documented (Meng and Takeichi, 2009; Waterman-Storer et al., 2000; Ren et al., 1999; Stehbens and Wittmann, 2012). Finally, the focal adhesion kinase (FAK) also influences cell adhesion, growth and migration. Its major role is to transmit extracellular signals involving integrins and to moderate cell adhesion and migration by modulating the rearrangement of actin filaments and microtubules (Mitra et al., 2005). FAK has been investigated in mature spheroids, where a role in tumour growth has been suggested (Tancioni et al., 2015). Few studies have investigated the effect of FAK inhibition in forming aggregates (Beck et al., 2014; Thakur et al., 2015).

The problem with most of these studies is: they rely on spheroid culture conditions, which provide heterogeneous aggregates varying in size and shape (Tzanakakis et al., 2001; Yoshii et al., 2011; Beck et al., 2014; Thakur et al., 2015). Hence, effects on aggregate morphology are essentially not quantifiable. Further, no detailed temporally resolved analyses of self-assembly processes have been performed.

Here, we investigated the impact of cellular processes like cell–cell, cell–ECM adhesion and growth on tissue integrity. We performed detailed analyses of the temporal dynamics during spheroid formation. We complement the understanding of adhesion on forming spheroids by studying the role of E-cadherin, the cytoskeleton, i.e. actin and microtubules, and the integrin downstream effector FAK. Scaling our results to the tissue level provides an excellent understanding of all aspects that contribute to tissue integrity.

## RESULTS

### Spheroid formation dynamics in three different cell lines

**Fig. 1.**
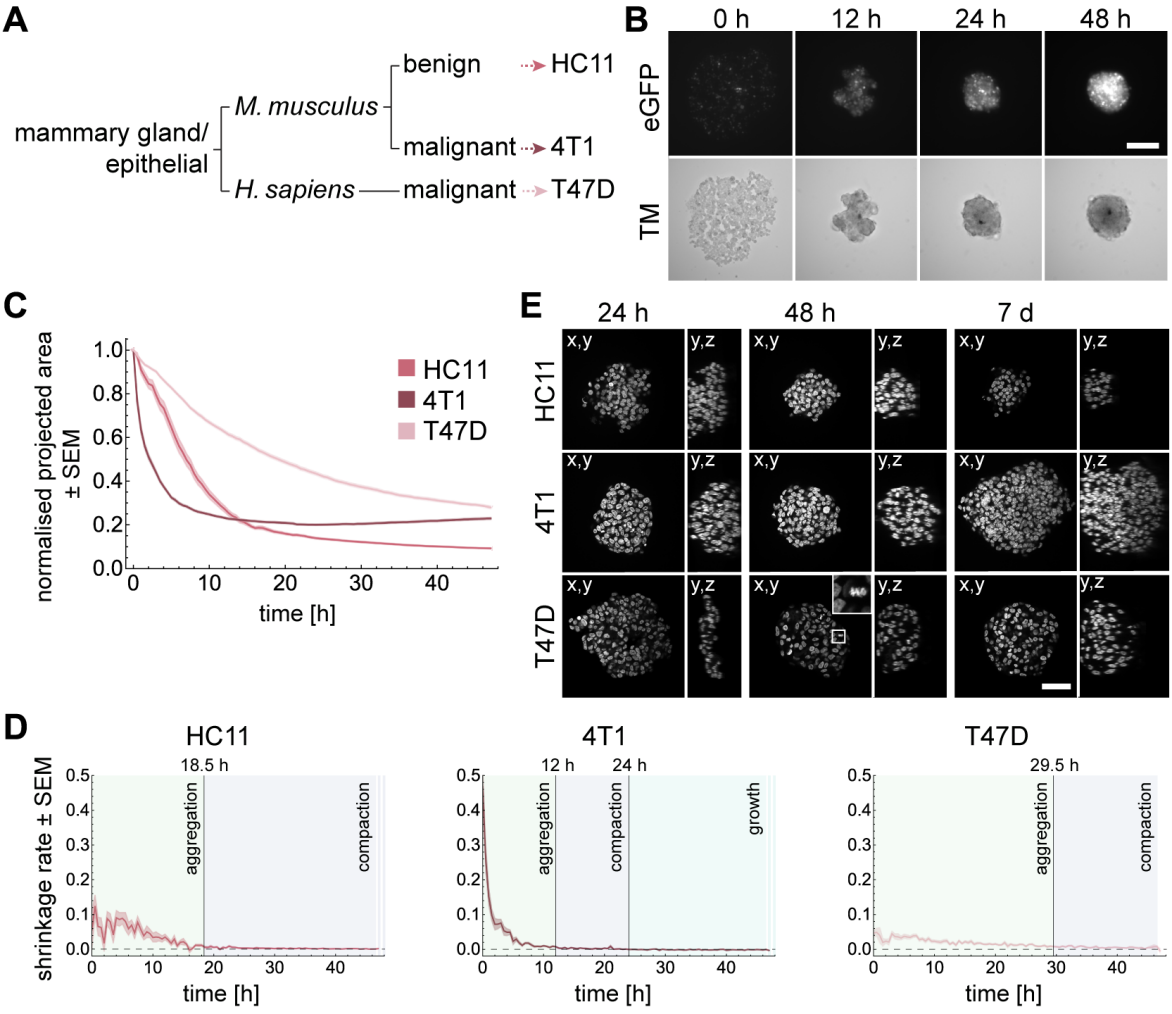
**Spheroid formation** **differs strongly** **between cell lines.** (A) Relationship between the cell lines regarding origin of tissue, species and phenotype. (B) Spheroid formation with HC11 cells for 48 h. Images show transmission channel and fluorescence channel of cell nuclei tagged with H2B-eGFP. Wide-field fluorescence microscopy: Carl Zeiss Cell Observer Z.1, objective: 10×/NA 0.5, time-lapse: 48 h, interval: 30 min, Scale bar: 50 µm. (C) Dynamics of the projected area occupied by HC11, 4T1 and T47D cells in DMSO normalised to the area at time 0 h. Shaded regions represent the standard error of the mean (SEM). Number of independent experiments for HC11, 4T1 and T47D cells: 30, 40, 30. (D) The shrinkage rate was approximated by ΔNA/Δt, where ΔNA is the difference of the normalised area between two time points and Δt the time step. The transition between aggregation and compaction phase is reached when the shrinkage rate drops below 0.008. The growth phase is reached when the shrinkage rate is below zero. (E) Views along different directions show spheroid dimensions at 24 h, 48 h and 7 days of formation. The 4× magnified section shows a mitotic cell. Microscope: Zeiss LSM780, objective: 40×/NA 1.3 oil, Scale bar: 50 µm.

To study the establishment of three-dimensional multicellular structures of different epithelial cell lines, we performed a spheroid formation assay. A highly invasive and metastatic mouse mammary epithelial cancer cell line (4T1) was used, which resembles late-stage human breast cancer (Heppner et al., 2000; Aslakson and Miller, 1992). We further used a human mammary epithelial cancer cell line (T47D), which is derived from a pleural metastasis and is classified as luminal A (ER^+^, PR^+^, Her2^−^) (Holliday and Speirs, 2011). The third cell line was the non-tumorigenic mouse mammary epithelial cell line HC11 (Ball et al., 1988). The cell lines differed in the origin of species and their metastatic potential, but were all of epithelial origin (Fig. 1A). The cell lines were modified to express eGFP-histone 2B and LifeAct-tagRFP. Spheroids were formed using the liquid overlay method (Carlsson and Yuhas, 1984). The dynamics of the aggregation process were followed for 48 h in a wide-field fluorescence microscope. To exclude effects from continuous light exposure, selected wells were only imaged at the beginning of the experiment and after 48 h.

To start the experiment, single cells were distributed in a non-adhesive environment (Fig. 1B, 0 h). During the time course, cells started to aggregate spontaneously (Fig. 1B, 48 h). A successfully formed spheroid is characterised by a compact, spherical shape.

To quantify the formation dynamics of the cells, the area occupied by the cells was measured over time (Fig. S1). The analysis of the area measurements revealed that the temporal dynamics of spheroid formation differed strongly between HC11, 4T1 and T47D cells (Fig. 1C). By computing the shrinkage rate, we identified the three phases of spheroid formation (Fig. 1D). In HC11 cells, the aggregation phase was completed after 18.5 h. Then, cells compacted until at least 48 h of spheroid formation. 4T1 cells aggregated within the first 12 h. The compaction was overcome by the beginning of growth after 24 h. Both, HC11 and 4T1 cells showed a discoidal shape of the aggregates after 24 h, which became spherical after 48 h (Fig. 1E). In T47D cells, the transition between the aggregation phase and the compaction phase was reached after 29.5 h (Fig. 1D). After 24 h, T47D cells formed an aggregate with the thickness of a few cells, which further thickened until 48 h (Fig. 1E). T47D cells aggregated much slower and did not even achieve the round shape of the 4T1 spheroids after 48 h.

To further investigate the aggregation dynamics of these cell lines, the impact of E-cadherin, actin, microtubules and FAK was assessed during the spheroid formation process. Since spheroid formation is a multi-step process, we focused on different time points: 6, 12, 24, 36 and 48 h following the initiation of formation. In addition, we measured the projected area of the spheroids after 7 days of formation to identify the impact of the different components on long-term growth. To examine the binding capacities during spheroid formation, we evaluated the experimental data with a previously developed three-dimensional agent-based computational model (Garg et al., 2015).

### E-cadherin is indispensable for spheroid formation

**Fig. 2.**
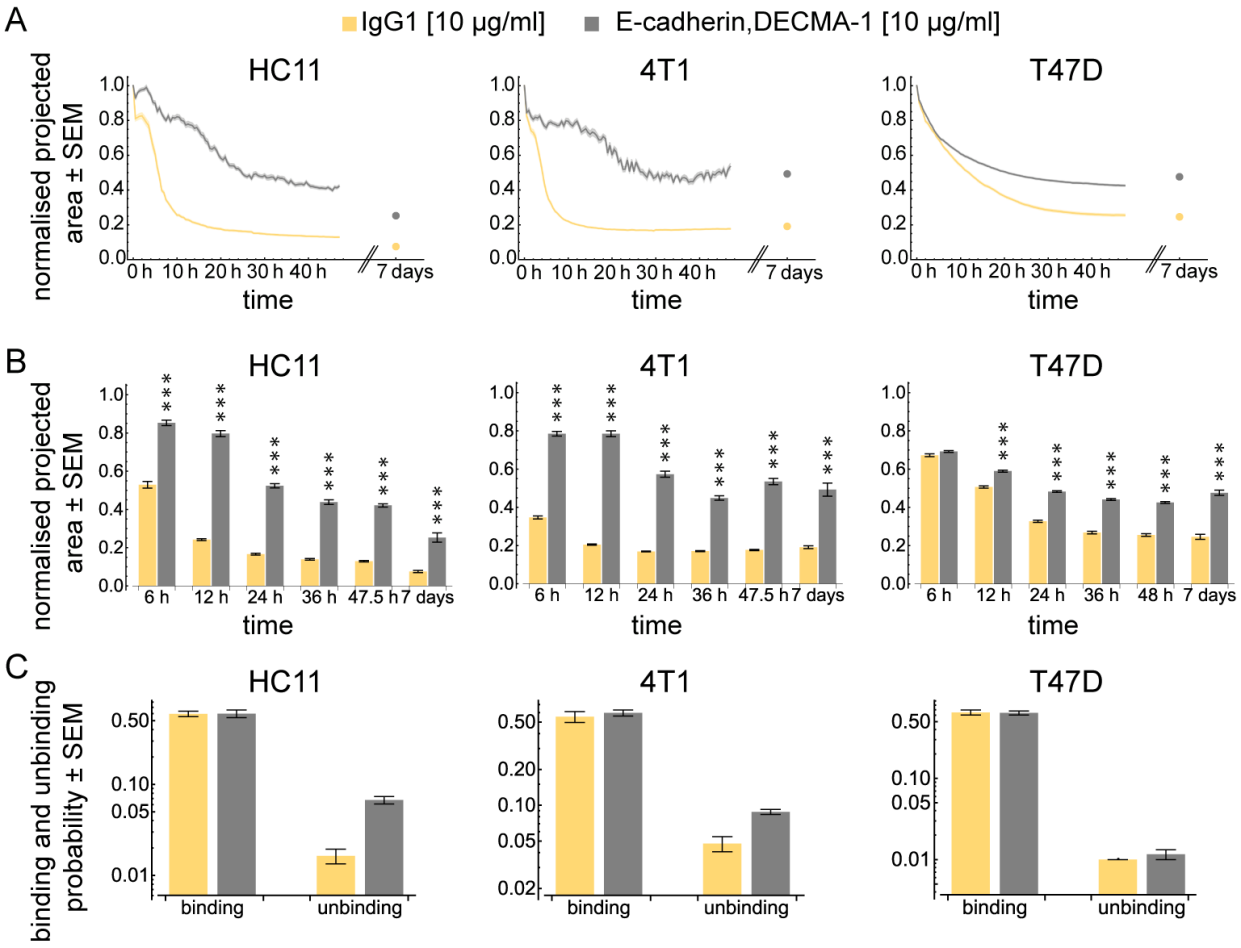
**E-cadherin is necessary to form spheroids.** (A) Dynamics of the projected area occupied by HC11, 4T1 and T47D cells normalised to the area at time 0 h. Shaded regions represent the standard error of the mean (SEM). Note that in some cases, due to the small error, the shaded region is not visible. Dots indicate the projected area after seven days. The antibody concentration for each cell line is indicated in brackets. (B) Bars show the normalised projected area after 6, 12, 24, 36, 48 h and after seven days for HC11, 4T1 and T47D cells. Hypothesis testing was performed using a Wilcoxon rank sum test with Holm correction for multiple testing. Asterisks indicate significant differences (*<0.05, **<0.01, ***<0.001). Number of independent experiments for HC11, 4T1 and T47D are summarised in Table S2. (C) Binding and unbinding probabilities obtained from fitting the computational model to the experimental data.

E-cadherin is an important mediator for cell–cell adhesion (Takeichi, 1991). To examine the role of E-cadherin, we inhibited its function with the antibody DECMA-1. The appropriate concentration was 10 µg/ml for each of the three cell lines. In line with previous studies (Ivascu and Kubbies, 2007; Lin et al., 2006), neither of the three cell lines were able to form spheroids upon E-cadherin function inhibition (Fig. 2A). The projected area of HC11 and 4T1 spheroids was significantly increased at each time point compared to the respective controls (Fig. 2B). T47D spheroids showed differences from 6 h onwards. The effects were not caused by continuous light exposure (Table S1). After 7 days, solely HC11 spheroids further reduced in size compared to the last time point of the time-lapse of the spheroid formation assay.

Interestingly, HC11 and 4T1 cells showed strong variations of the projected area over time. These fluctuations were not observed in T47D aggregates (Fig. 2A and Fig. S2). To further investigate the binding properties of the cells, we fitted the computational model to our data. We obtained quantitative information about the probability, at which two cells bind or unbind. Interestingly, the computational model revealed that a block of E-cadherin function strongly increased the unbinding probability in both, HC11 and 4T1 cells, while the binding probability was not affected (Fig. 2C and Fig. S3). Hence, E-cadherin is rather involved in stabilising already existing contacts but not for contact building. In T47D cells, neither the binding nor the unbinding probability was affected when E-cadherin function was disabled (Fig. 2C and Fig. S3).

### An intact actin cytoskeleton is indispensable for spheroid formation

Although the actin cytoskeleton is known to play a role in adhesion and further mediates cell shape, spreading and migration (Tzanakakis et al., 2001; Yoshii et al., 2011; Gardel et al., 2010), relatively little is known about the role of actin in contact formation during spheroid formation across different cell types. We used cytochalasin D to block the polymerisation at the growing end of actin filaments (Fox and Phillips, 1981).

**Fig. 3.**
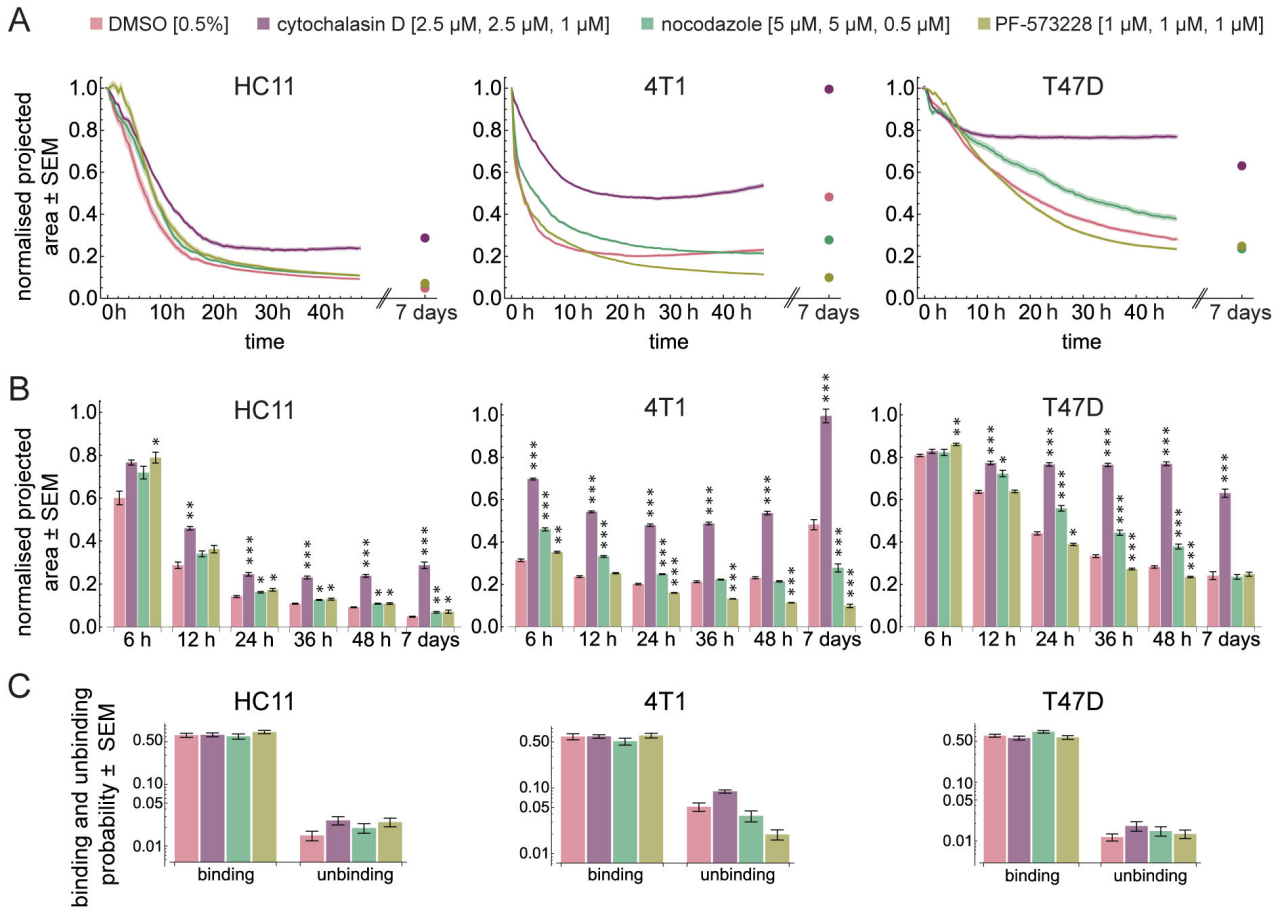
**Spheroid formation dynamics.** (A) Dynamics of the projected area occupied by HC11, 4T1 and T47D cells normalised to the area at time 0 h. Shaded regions represent the standard error of the mean (SEM). Dots are the projected area measured after seven days. (B) Bars show the normalised projected area after 6, 12, 24, 36, 48 h and after seven days in HC11, 4T1 and T47D cells. Hypothesis testing was performed using a Wilcoxon rank sum test with Holm correction for multiple testing. Asterisks indicate significant differences (*<0.05, **<0.01, ***<0.001). Different drugs were compared against the DMSO control. Number of independent experiments for HC11, 4T1 and T47D are summarised in Table S2. (C) Binding and unbinding probabilities obtained from fitting the computational model to the experimental data.

In the presence of cytochalasin D, the aggregation was strongly reduced (Fig. 3A, purple; Fig. S4). All cell lines showed a significantly larger projected area (12, 24, 36, 48 h and 7 days) compared to the respective control (Fig. 3B). Especially, T47D cells stopped aggregating already after about 10 h. Although HC11 and 4T1 cells aggregated slowly until at least 20 h or 10 h, respectively, they failed to form spheroids after 48 h (Fig. S4). The observed effects were not caused by continuous light exposure (Table S1).

After 7 days, cytochalasin D-treated spheroids were fragile while pipetting, indicating that no proper connection between cells was formed. After 7 days of spheroid formation, cytochalasin D-treated 4T1 spheroids showed an increased projected area. This was presumably due to an increased cell nuclei volume (Fig. S5). After 7 days, the projected area of HC11 spheroids was slightly increased compared to the last time point of the time lapse, whereas T47D spheroids further compacted (Fig. 3A, purple; Fig. S4).

According to the computational model, the probability at which two cells bind decreased slightly (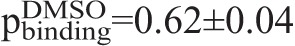, 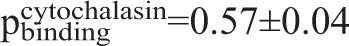) in T47D cells upon cytochalasin D treatment, whereas it was not altered in HC11 and 4T1 cells (Fig. 3C; Fig. S3). The unbinding probability was at least doubled in all cell lines. This indicates that connections are less stable upon the loss of an intact F-actin network.

Together, our data show that the actin cytoskeleton is a crucial factor driving aggregation of isolated cells in different cell lines. The results from our simulations suggest that this is due to a stabilising effect of connections.

### Depolymerisation of microtubules primarily decelerates the aggregation and compaction of spheroid formation

Next, we formed aggregates in the presence of nocodazole, which interferes with the polymerisation of microtubules (Samson et al., 1979).

Nocodazole slightly impaired the compaction phase in the non-tumorigenic mammary epithelial cell line HC11. Within the first 12 h of spheroid formation, we observed a decelerated aggregation. However, this was not significantly different from the control. After 24 h, HC11 spheroids were less compact if cells were treated with nocodazole. After 7 days, HC11 spheroids were not as dense as the controls (Fig. 3A and B, HC11 green; Fig. S4). The aggregation of 4T1 cells treated with nocodazole significantly slowed down during the initial 24 h of spheroid formation compared to the control (Fig. 3A and B, 4T1 green; Fig. S4). However, cells still formed a compact spheroid after 48 h. After 7 days, 4T1 spheroids showed an increased size compared to the last time point of the time lapse. However, those spheroids did not reach the size of the controls. For T47D cells, spheroid formation decelerated from 12 h onwards. After 7 days, the sizes of the spheroids treated with nocodazole were comparable to the sizes of the control spheroids.

To further investigate the deceleration of spheroid formation, we considered the binding and unbinding probability of nocodazole treated cells, and compared it to the control. Spheroids formed from either HC11 or 4T1 cells showed an unaltered binding probability, while the unbinding probability was increased (HC11: 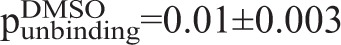, 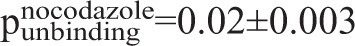, 4T1: 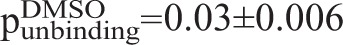, 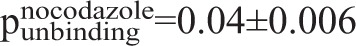) when cells were treated with nocodazole. Interestingly, although the formation dynamics were decelerated in T47D cells, their binding probability was increased (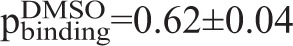, 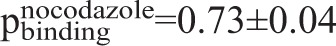), while the unbinding probability was not changed (Fig. 3C; Fig. S3).

Together, these data show that microtubules participate the aggregation and compaction and growth phases of spheroid formation. Dependent on the cell line, microtubules stabilise contacts or counteract the reinforcement of adhesion. Although spheroid formation was decelerated, cells retained the ability to aggregate to a certain extent.

### Inhibition of FAK activity affects all three phases in a cell type specific manner

Integrins have been described in the context of spheroid formation (Ivascu and Kubbies, 2007; Lin et al., 2006). To study the importance of signal transduction from the outside to the inside of a cell, we inhibited the phosphorylation of FAK at the position Y397 with the inhibitor PF-573228 (Slack-Davis et al., 2007).

In all three cell lines, the initial aggregation phase during the first 6 h showed an increased projected area compared to the respective controls. However, after 24 h the behaviour of all cell lines was altered differently. Spheroid compaction decelerated in HC11 spheroids. After 7 days, FAK inhibition in HC11 spheroids increased the projected area compared to the control (Fig. 3A and B, HC11 olive; Fig. S4). The growth of FAK inhibitor-treated 4T1 spheroids was impaired (Fig. 3A and B, 4T1 olive; Fig. S4). From 24 h onwards, T47D spheroids were smaller compared to the control. After 7 days, no difference was measured compared to the control (Fig. 3A,B, T47D olive).

The binding probability obtained from the computational model was slightly increased in HC11 cells treated with PF-573228 compared to the control (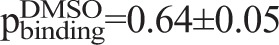, 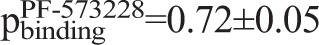) (Fig. 3C; Fig. S3). The probability to break cell contacts was doubled in HC11 cells (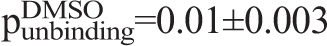, 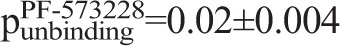). In T47D cells, neither the binding nor unbinding probabilities were altered when cells were treated with PF-573228. Interestingly, although PF-573228 showed a strong effect in the spheroid formation of 4T1 cells, we observed a slight effect on either, the binding or the unbinding probability (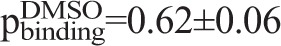, 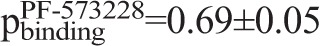 and 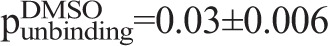, 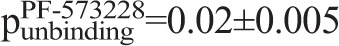).

These data suggest that FAK activity affects each phase of spheroid formation in a cell type dependent manner. Nevertheless, spheroids form to a certain extent, which indicates that spheroid formation does not rely on intact FAK signalling.

### Drug treatment effects on cell death during spheroid formation and cell viability

Since both nocodazole and PF-573228 affected the size of HC11 and 4T1 spheroids, we investigated whether the cell viability was influenced by drug treatment. In monolayer cultures, we found that a 24 h incubation with cytochalasin D or nocodazole decreased cell viability in HC11 and 4T1 cells. Cytochalasin D treatment increased the cell viability in T47D cells. Interestingly, the FAK inhibitor PF-573228 only affected cell viability in HC11 cells, indicated by an increase of 42.5% (Fig. S6).

### The difference in aggregation and compaction is mainly due to differences in adhesion capability on ECM components and in the arrangement of ECM

The inhibition of FAK phosphorylation revealed a cell type dependent role of FAK in spheroid formation. Thus, we further investigated the details of this finding.

**Fig. 4.**
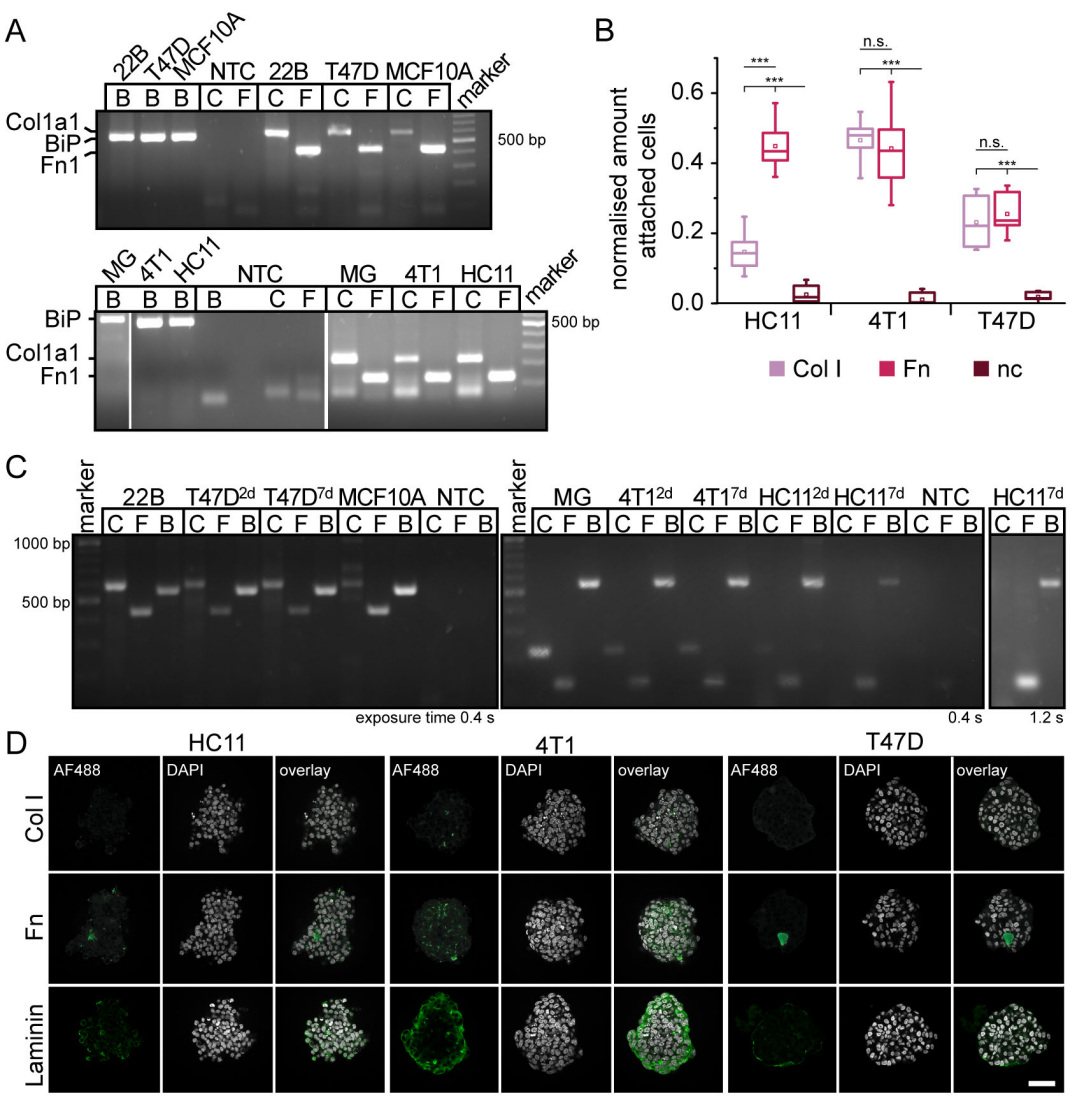
**Extracellular matrix is expressed in cellular spheroids.** (A,C) Amplification of human and murine Col1a1 and Fn1 mRNA. Amplicon length of human and mouse Col1a1 is 600 bp and 223 bp, respectively. Amplicon length of human and mouse Fn1 is 438 bp and 124 bp, respectively. BiP amplification was used as a control and had a fragment size of 560 bp. As positive control, RNA from murine mammary glands (MG), MCF10A epithelial breast cells and 22B endometriotic stromal cells was used. (A) RNA was extracted from cell lines in monolayer culture, or (C) from spheroids cultured for two or seven days. (B) Cell lines showed different behaviours in attaching to surfaces coated with ECM-components. (D) ECM components are expressed in spheroids within 24 h of formation. The different matrices show different patterns in the spheroids. Microscope: Zeiss LSM780, objective: 40×/1.3 oil, Scale bar: 50 µm. Col I, collagen I; Fn, fibronectin; B, BiP; C, collagen 1a1; F, fibronectin 1.

FAK is involved in transmitting signals initiated by integrin-ECM interactions into the cell (Schlaepfer et al., 1999). Therefore, we analysed whether HC11, 4T1 and T47D cells expressed ECM proteins such as collagen I or fibronectin *in vitro*. We found that all cell lines expressed collagen 1a1 and fibronectin 1 mRNA in monolayer cultures (Fig. 4A). We further investigated the adhesion capability of the cells in an adhesion assay. The three cell lines attached to both, fibronectin 1- and collagen I-coated surfaces. Interestingly, the number of attached cells differed between cell lines as well as among the two matrix components. An increased number of HC11 cells bound to fibronectin 1 compared to collagen I. Both 4T1 and T47D cells bound equally well to either collagen I or fibronectin 1. However, T47D cells generally showed a reduced attachment compared to 4T1 (Fig. 4B).

Next, we investigated whether ECM proteins are expressed in the spheroids. Thus, we extracted RNA from spheroids after 48 h and after 7 days of spheroid formation to detect collagen 1a1 and fibronectin 1. We found that fibronectin was expressed in spheroids of all cell lines at both time points. Collagen 1a1 mRNA was expressed in T47D and 4T1 cells at both time points. In HC11 cells, we detected collagen 1a1 mRNA only after 48 h of spheroid formation but not in spheroids formed for 7 days (Fig. 4C). We complemented this finding by detecting collagen I, fibronectin and laminin in spheroids by an immunofluorescence staining (Fig. 4D). The fibronectin antibody may show both, plasma and cellular fibronectin.

These data prove that cellular spheroids produce ECM. This and the capability of cells to differently adhere to the ECM may have an influence on the aggregation and compaction of spheroids.

### FAK activity is not required for anchorage-independent spheroid growth and further correlates with invasiveness of cells

Nuclear localisation of FAK alters gene expression and thereby influences spheroid growth independently from anchorage and an association with integrins (Lim, 2013; Tancioni et al., 2015). To analyse whether this integrin-independent mechanism was effective in our spheroids, we immunolabelled FAK in spheroids formed for 48 h. In all three cell lines, FAK exhibited a cytoplasmic distribution (Fig. S7), diminishing the possibility of an integrin-independent influence of FAK on spheroid growth.

**Fig. 5.**
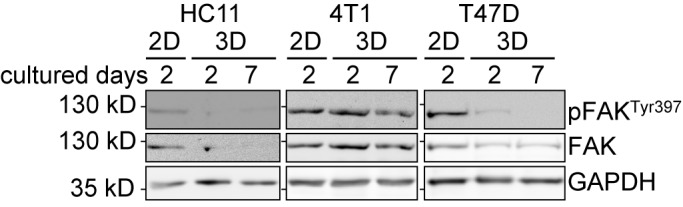
**FAK expression and FAK phosphorylation differ between cell lines and culturing methods.** Immunoblot analysis was performed with protein extracts from monolayers and spheroid cultures. Cells were lysed in RIPA buffer and analysed by SDS-PAGE. Antibodies against FAK and pFAK^Tyr397^ were used. GAPDH is shown as the loading control. Cell lysates from different cell lines were loaded onto different gels. Protein lysates from all conditions of a cell line were loaded onto the same gel. Uncropped data is shown in Fig. S8, quantitative densitometry data is shown in Table. S3.

It has been reported that the expression and activity of FAK differs depending on the grade of malignancy (McLean et al., 2005). Therefore, we analysed protein extracts from spheroids grown for 2 days and 7 days to detect the protein level and phosphorylation of FAK. FAK activity heavily depends on integrin signalling. Since integrins are expressed differently in cells grown in two- versus three-dimensional cultures (Cukierman et al., 2001), we extracted proteins from both, monolayers and spheroid cultures. We found that the protein levels of FAK and pFAK^Y397^ varied between cell lines and also between monolayer and spheroid culture. In HC11 cells, FAK protein level was reduced to the detection minimum in spheroids, while both, FAK and pFAK^Y397^ were detectable in monolayer cultures (Fig. 5, left column). Interestingly, the signal of either FAK or pFAK^Y397^ was strong in 4T1 cells and similar between two- and three-dimensional cultures (Fig. 5, middle column). For T47D cells, the amount of pFAK^Y397^ differed between monolayer culture and spheroids. While pFAK^Y397^ was detectable in the monolayer culture, its detection was weak in spheroids cultured for 2 days and it was not detectable at 7 days of spheroid culture. In all conditions, a basal level of FAK was detectable (Fig. 5, right column).

These results show that FAK protein levels strongly differ between two- and three-dimensional cultures and that FAK phosphorylation is differently regulated among different cell lines.

## DISCUSSION

Tissue integrity maintains the functionality of a tissue through a tight regulation of cell–cell and cell–ECM adhesion as well as growth. These cellular processes have been intensively studied in cells grown on rigid, two-dimensional surfaces. Many differences between two-dimensional and more physiological three-dimensional cell cultures have been documented in the literature (Pampaloni et al., 2007, 2010). Cells grown in a monolayer culture form extensive numbers of focal adhesions, which can have antagonising effects on the formation of cell–cell adhesion sites and cell migration (Cukierman et al., 2001). Thus, the molecular mechanisms are strongly influenced by non-physiological culture conditions.

The mechanisms that facilitate adhesion in three-dimensional cell aggregates as well as in tissues are largely unknown. Hence, we studied the contributions of different adhesion-associated proteins in a spheroid formation assay.

The aggregation of cells into multicellular structures in a non-adhesive environment has been observed for various cell types. Several studies have described the kinetics of spheroid self-assembly. Neelamegham and colleagues found that the aggregation mechanism is primarily diffusion-controlled, i.e. cells almost always adhere upon collision (Neelamegham et al., 1997). Conflictingly, Enmon et al. proposed a reaction-controlled aggregation. Here, adhesion does not necessarily occur when two cells collide (Enmon et al., 2001). We employed our recently developed three-dimensional computational model for spheroid formation (Garg et al., 2015). Fitting the model to our experimental data revealed a binding probability of at most 73% for each condition. This suggests that the spheroid formation process is reaction- rather than diffusion-controlled. Hence, aggregation is an active process, in which cells get in contact upon collision, but do not necessarily form connections.

Previous studies have suggested a general mechanism for spheroid formation (Lin and Chang, 2008; Enmon et al., 2001). We successfully identified the three phases of spheroid formation. They comprise an initial aggregation, compaction and spheroid growth. However, phase transitions do not occur abruptly. The characterisation of a phase means that a certain process dominates this phase. For example, cell division takes place at any time during the formation process (see Fig. 1E, T47D 48 h, magnified section), but it does not dominate during the aggregation and compaction phases. With our image-based spheroid formation assay, we showed that spheroid formation differs substantially amongst the three investigated cell lines: 4T1 aggregated the fastest, followed by HC11, and T47D aggregated by far the slowest. Since no obvious explanation for the observed differences can be based on the invasive phenotype of the cells, we further investigated the role of different proteins that are associated with adhesion during spheroid formation. Therefore, we inhibited E-cadherin, the actin and microtubule networks as well as FAK.

Cadherins are essential mediators for cell–cell contacts and crucial for a successful spheroid formation (Ivascu and Kubbies, 2007; Lin et al., 2006; Saias et al., 2015). Like previous studies, our results showed that spheroid formation was impaired when E-cadherin was blocked. Moreover, the computational model revealed that the inhibition of E-cadherin has no influence on the binding probability of the cells. This indicates that the presence of E-cadherin does not increase the chance of forming cell contacts when cells collide. Instead, other adhesion-facilitating proteins may be crucial for this process. A previous study has found that desmosomal proteins are important for spheroid formation (Saias et al., 2015). To which extent they influence the binding probability of cells remains elusive. In two out of the three cell lines (HC11 and 4T1 cells), the unbinding probability was strongly increased when E-cadherin was blocked. This suggests that E-cadherin contributes to stabilising cell contacts. This is further supported by the occurrence of strong fluctuations of the projected area during spheroid formation, which are caused by fragile connections between the cells. This result provides a yet unknown aspect of E-cadherin in the three-dimensional cellular context.

Although the actin cytoskeleton is crucial in spheroid formation details have never been investigated. Consistent with previous studies (Tzanakakis et al., 2001; Yoshii et al., 2011), we showed that the actin cytoskeleton is indispensable for spheroid formation. Cytochalasin D disrupted the F-actin network and affected the self-assembly of the cells. Cells did not form proper spheroids, but clusters with loose cell contacts.

We further analysed the dynamics of spheroid formation of cytochalasin D-treated cells computationally. The binding probability was mainly unaffected, suggesting that actin is less important for cells to form a connection. The increased unbinding probability in all cell lines suggests that cells tend to lose contacts when the actin cytoskeleton is disrupted. Hence, a role of the actin cytoskeleton during spheroid formation is to reinforce contacts.

Upon cytochalasin D treatment of 4T1 cells, we observed a continuous increase in spheroid size and also an increase in cell volume. The actin cytoskeleton is involved in cell volume regulation (Mills et al., 1994) and the disruption of actin filaments abolishes this process (Blase et al., 2009). Although HC11 cells were treated at the same drug concentration, no cell swelling was observed, showing that cell swelling is cell type specific (Pedersen et al., 2001).

The role of microtubules in cell aggregation has been investigated only rarely and no quantitative analysis of spheroid formation dynamics has been performed. Both effects, an inhibition of spheroid formation and no impact on spheroid formation upon microtubule impairment, have been described (Tzanakakis et al., 2001; Yoshii et al., 2011). We showed that nocodazole-treated cells sustain the ability to form spheroids. Our experiments provided detailed and temporally resolved results, which show that the formation is slowed down upon nocodazole treatment in all cell lines.

The growth phase in 4T1 spheroids was inhibited by a depolymerisation of microtubules in the long term. Further, the cell viability was markedly reduced.

According to the computational model, the binding and unbinding probabilities were differently affected in the three cell lines upon nocodazole treatment. In HC11 and 4T1 cells, the binding probability was not affected, but the unbinding probability was increased. This indicates that microtubules are important for the stabilisation of already formed contacts. Microtubule-mediated vesicle transport is necessary to translocate adhesion molecules to the plasma membrane (Mary et al., 2002). We conclude that upon microtubule destabilisation, cells fail to accumulate adhesion molecules at the plasma membrane to reinforce connections. This leads to the decreased spheroid formation speed.

In T47D cells, the aggregation speed was similarly impaired compared to the other cell lines. The computational model revealed that the unbinding probability was not affected. T47D cells exhibit strong cell–cell contacts and strongly express adhesion molecules at the surface (Holliday and Speirs, 2011; Ivascu and Kubbies, 2007). Consequently, transport of adhesion-associated proteins to the plasma membrane might not be that important in these cells. Moreover, the binding probability was increased when microtubules were depolymerised. This strongly suggests that single cells assemble and form contacts in one plane at the bottom of the wells. These strong cell contacts restrict the cells ability to move on top of each other against gravity. Consequently, cells are unable to arrange into the third dimension (i.e. into a proper, spherical spheroid). This agrees well with the increased projected area of nocodazole-treated cells during spheroid formation. The correlation of binding probability and microtubule depolymerisation might be due to the influence of microtubules on the activity of E-cadherin. A recent study has unveiled that a depolymerisation of microtubules activates E-cadherin by mediating the dephosphorylation of p120-catenin (Maiden et al., 2016). From this, we draw the conclusion that an intensified contact formation negatively affects the rearrangement of cells to move on top of each other. It further shows that microtubules are important to maintain a balance between formation and the rearrangement of contacts.

A recent study has described the importance of FAK phosphorylation at Y397 during spheroid formation of human dental follicle cells. Spheroid formation has been induced by culturing the cells under serum-free medium culture conditions on dishes coated with poly-L-lysine (Beck et al., 2014). Thus, a large portion of these cellular aggregates remained attached to the surface of the dish, which understandably results in FAK phosphorylation events caused by integrin adhesion to the coated surface. Our approach circumvents the attachment to any non-natural surface and provides controlled and true three-dimensional culture conditions.

We observed that the spheroid formation process itself is not heavily affected by FAK inhibition in all cell lines. However, our results showed cell type specific effects. The adhesion to the substratum activates FAK (Mitra et al., 2005). An increased expression of FAK and its activity is associated with tumours (McLean et al., 2005). We found that the basal protein level as well as the tyrosine phosphorylation at position 397 varies between the cell types. In HC11 and T47D cells, FAK phosphorylation decreased in spheroids compared to monolayer cultures, which indicates that integrin-mediated signalling via FAK is less important in non-invasive cells in three-dimensional aggregates. In line with this, HC11 spheroid formation was also found to be the least impaired. Conversely, the highly invasive 4T1 cells showed a strong effect when FAK was inhibited. These cells showed high FAK phosphorylation in spheroids. Consequently, FAK activity correlates with the invasiveness of cells and influences growth only if a high amount of active FAK is present.

FAK is essential for tumour growth (Wendt et al., 2011; Tanjoni et al., 2010; Tancioni et al., 2015). The influence of FAK on cell proliferation can occur in an integrin-dependent manner but also independent from integrin-ECM association (Lim, 2013). Integrin-independent effects on proliferation exhibit a nuclear localisation of FAK. In the spheroids we did not observe a nuclear translocation of FAK. Thus, we suggest that the observed effects on spheroid growth originate from integrin-mediated signalling.

4T1 spheroid growth is reduced upon FAK inhibition (Tanjoni et al., 2010), which is consistent with our observations. The strong reduction in growth upon FAK inhibition in 4T1 spheroids might be due to the strong FAK activity in these cells as indicated by a high phosphorylation at Y397 in untreated spheroids. We could not observe an altered cell viability in 4T1 monolayer cultures, which is consistent with previous findings (Tanjoni et al., 2010). This demonstrates that cells can show opposite effects when cultured in a two-dimensional monolayer compared to three-dimensional culture.

The computational model fitted to our experimental data showed that pharmacological inhibition of pFAK^Y397^ influenced the binding as well as the unbinding probability in all three cell lines differently, underpinning the assumption that cell aggregation is differently influenced by FAK in different cell types. This can have two possible causes: (1) FAK activity affects cytoskeleton remodelling (Mitra et al., 2005), or (2) FAK modulates cell–cell adhesion (Koenig et al., 2006; Playford et al., 2008; Yano et al., 2004).

The aggregation of cells is mediated by cell–cell adhesion and may be influenced by cell–ECM adhesion. The spheroids formed from the three cell lines expressed ECM proteins such as collagen I and fibronectin, which is in line with previous studies (Bjerkvig et al., 1989). In addition, we showed that the expression of ECM proteins occurs very early in the forming spheroids (during the first 24 h). The severity of this effect may depend on the general ability of the cell to associate with the ECM. We showed that the capability of the cells to attach to ECM differs between cell lines. It further indicates that cells not only aggregate by spontaneous cell–cell attachment, but also by cells incorporating ECM into the aggregate, which may further facilitate spheroid formation.

**Fig. 6.**
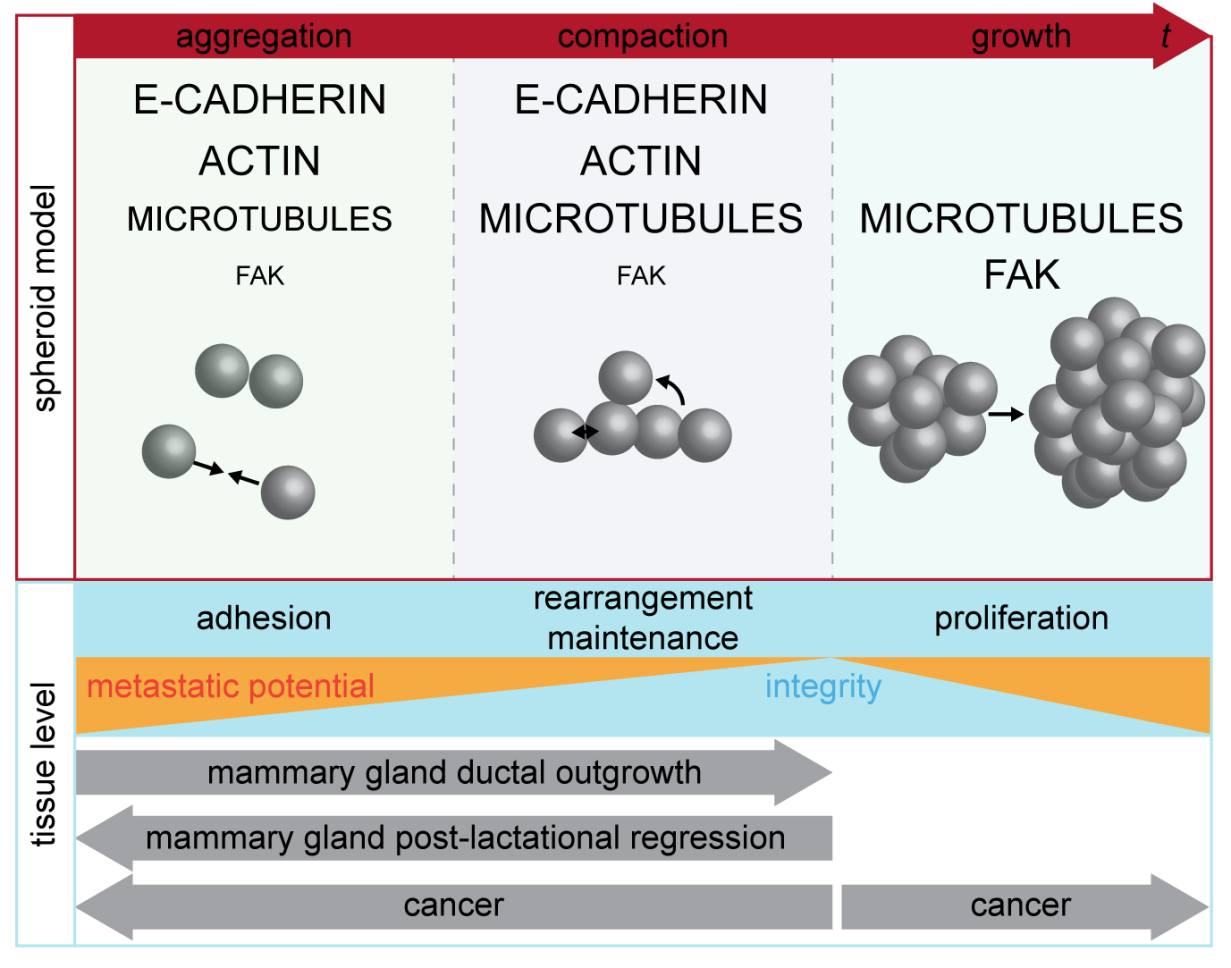
**Model showing the phase-dependent roles of intracellular components in three-dimensional cell aggregations.** Spheroid formation on a non-adhesive surface is divided in the three phases: aggregation, compaction and growth. The involvement of the three intracellular components E-cadherin, actin, microtubules and FAK is indicated for each phase. The size of the capital letters indicates the importance in the according phase. The phases of spheroid formation are extrapolated to the tissue level. The tissue integrity is high when cellular rearrangement and maintenance of adhesion by reinforcement occur. Uncontrolled proliferation counteracts tissue integrity and correlates with the metastatic potential. Developmental processes *in vivo*, such as mammary gland development and cancer development, follow both directions of an integrated tissue.

We dissected spheroid formation into three temporally distinct phases. For these phases, we identified the importance of the four proteins E-cadherin, actin, microtubules and FAK (Fig. 6). We conclude that spheroid formation is a process that is not solely driven by cell–cell adhesion but also by the rearrangement of the cells, which might be driven by migration on the ECM.

We propose that the phases, which are present during spheroid formation, can be extrapolated to the tissue level. Tissue integrity increases if adhesion is facilitated, if the cells rearrange to their determined position or if adhesion contacts are maintained by reinforcement. Depending on the developmental needs, tissue integrity may increase or decrease. Mammary gland ductal outgrowth during puberty is directed towards an integrated tissue, whereas post-lactational regression disintegrates the tissue to accomplish remodelling processes. In cancer, increasing metastatic potential counteracts tissue integrity by destabilisation of adhesion and uncontrolled proliferation (Fig. 6).

Our previous and current studies contribute to an overall picture of cellular aggregates. A physiological understanding of cell adhesion and growth can only be achieved with three-dimensional dynamic models. Our results encourage future research on how tissues develop, how they are maintained and what causes their disruption.

## MATERIALS AND METHODS

### Cell culture

T47D (HTB-133, ATCC) and 4T1 (CRL-2539, ATCC) cells were cultured in RPMI 1640 supplemented with 2 mM L-glutamine and 10% FBS. HC11 (kind gift from B. Groner, Georg-Speyer-Haus Frankfurt, Germany) cells were maintained in RPMI 1640 supplemented with 2 mM L-glutamine, 10% FBS, 5 µg/ml human recombinant insulin and 10 ng/ml murine EGF (Peprotech). Cells were maintained in their respective medium at 37°C and 5% CO_2_. No further authentication was performed for the cell lines. For experiments, assay medium consisting of RPMI 1640, 2 mM L-glutamine and 10% FBS was used for all cell lines. All cell lines were tested mycoplasma negative.

### Transgenecity

The H2B coding sequence was cloned into the lentiviral shuttle vector pLeGO-iG2 (Weber et al., 2008) to the fluorescent protein eGFP. In a second step, LifeAct-tagRFP was cloned between the SFFV (spleen focus-forming virus) promoter and the IRES (internal ribosomal entry site) sequence. 4T1 and HC11 cells were transduced with viral particles in the presence of 8 µg/ml polybrene (Sigma-Aldrich) in growth medium. Transgenecity of T47D cell (RCASΔF1-H2B-eGFP and RCASΔF1-LifeAct-tagRFP) was performed using the RCAS-TVA gene transfer system (Hughes, 2004). Fluorescent cells were enriched using fluorescent-activated cell sorting (FACS).

### Spheroid formation

The liquid-overlay technique was used for spheroid formation (Carlsson and Yuhas, 1984). Briefly, 96-well culture dishes were coated with 50 µl 1% low-melt agarose to form concave non-adhesive wells. Per well, 600 cells were seeded in 50 µl assay medium containing 0.5% penicillin/streptomycin. Drugs were diluted to their respective dilution and 50 µl drug solution was added to each well. Cytochalasin D (Enzo), nocodazole (Sigma-Aldrich), and PF-573228 (Sigma-Aldrich) were dissolved in DMSO (Sigma-Aldrich) with a final concentration of 0.5%. For all drugs, the appropriate, non-toxic concentration for each cell line was determined beforehand.

To inhibit E-cadherin, the DECMA-1 antibody (ab11512, Abcam) or the control IgG1 antibody (ab18407, Abcam) were used at 10 µg/ml. Well plates were centrifuged at 400 ***g*** for 4 min and then subjected to further analyses.

### Cell adhesion assay

Wells of a 96-well plate were coated with 2 µg bovine fibronectin (Sigma-Aldrich), 5 µg bovine collagen I (Gibco), or were left uncoated. Free binding sites were blocked with BSA. Hoechst 33342-stained (Life Technologies) cells were seeded at 1×10^5^ cells per well and incubated for 1 h at culture conditions. Non-adherent cells were washed off and fluorescence intensity of attached cells was measured with the microplate reader Infinite M200 (Tecan).

### Cell viability assay

7500 cells per well were seeded into wells of a 96-well plate and grown for 18 h. Then, cells were treated with drugs at the concentrations used during the spheroid formation assay for 24 h. Subsequently, 20 µl MTS solution (Aqueous One Solution, Promega) were added and cells were incubated for further 2–4 h. Absorbance at 490 nm and background at 700 nm were measured with the microplate reader Infinite M200 (Tecan).

### Western blot analysis

Cells grown as monolayer culture and spheroids were lysed by adding lysis buffer (0.5% sodium deoxycholate, 1% NP-40, 0.1% sodium dodecyl sulfate), 1 mM EDTA in PBS, and freshly added protease inhibitors (Sigma-Aldrich) and phosphatase inhibitors (Merck) and incubated for 20 min at 4°C. Lysates were sonicated (UP50H, Hielscher) for 20 s and centrifuged at 10,000 ***g*** for 15 min at 4°C.

Proteins were resolved on SDS-polyacrylamide gels, and transferred onto nitrocellulose membranes (GE Healthcare). Primary antibodies against GAPDH (1:10,000, AM4300, Ambion), FAK (1:1000, 610088, BD Biosciences), or pFAK^Tyr397^ (1:500, 3283, Cell Signaling Technology) were incubated over night at 4°C. Secondary horseradish peroxidase-conjugated antibodies (1:30,000 for 115-035-003, 1:10,000 for 111-035-003, Jackson ImmunoResearch) were incubated for 1.5 h at room temperature. Protein bands were visualised with an enhanced luminescence detection reagent with the Chemocam documentation system (Intas).

### Detection of ECM expression with polymerase chain reaction

Total RNA was isolated using TriZol (Life Technologies) or the NucleoSpin RNA kit (Macherey-Nagel). 1 µg RNA was reverse transcribed in a mix containing Maxima reverse transcriptase, dNTPs, oligo (dT)_18_ and random hexamer primers in a reaction buffer (Thermo Fisher Scientific). Reverse transcription was performed by incubating the sample first at 25°C for 10 min followed by an incubation at 50°C for 20 min and a heat inactivation at 85°C for 5 min.

Polymerase chain reaction on cDNA was performed using the Phusion polymerase (NEB). Mouse primers for fibronectin 1 and collagen I were the following: forward, 5′-ATGTGGACCCCTCCTGATAGT-3′, and reverse, 5′-GCCCAGTGATTTCAGCAAAGG-3′, and forward, 5′-CCTGGTAAAGATGGTGCC-3′, and reverse, 5′-CACCAGGTTCACCTTTCGCACC-3′, respectively. Human primer for fibronectin 1 and collagen I were the following: forward, 5′-CCGTGGGCAACTCTGTC-3′, and reverse 5′-TGCGGCAGTTGTCACAG-3′, and forward, 5′-TGACGAGACCAAGAACTG-3′, and reverse 5′-CCATCCAAACCACTGAAACC-3′, respectively.

### Immunofluorescence staining

Immunofluorescence staining of spheroids *in toto* was performed according to Smyrek and Stelzer (2017). The primary antibodies were anti-collagen I (1:100, ab-34710, Abcam), anti-fibronectin (1:100, ab-23750, Abcam), anti-laminin (1:100, L9393, Sigma-Aldrich), and anti-FAK (1:100, 610088, BD Biosciences) and were incubated over night at 37°C. The secondary antibodies were anti-mouse Alexa Fluor 568 (1:400, A10037, Molecular Probes) and anti-rabbit Alexa Fluor 488 (1:400, A11008, Molecular Probes) and were incubated for 4 h at 37°C. Cell nuclei were counterstained with 1 µg/ml DAPI (Thermo Fisher Scientific).

### Wide-field fluorescence microscopy

Time lapse data was recorded with the Cell Observer Z.1 (Carl Zeiss) for a duration of 48 h with 30 min intervals. Incubation conditions of 37°C and 5% CO_2_ were maintained during the acquisition period. A 10×/NA 0.5 objective (Carl Zeiss) was used. Fluorescence images (488 nm laser) and transmission images were acquired. Controls were imaged only at the beginning and the end of the time lapse to control effects caused by the light exposure (Table S1).

### Confocal laser scanning microscopy

Immunostained spheroids were mounted in a drop of Mowiol on a cover glass and image stacks were acquired with a 2 µm spacing in a Zeiss LSM780 confocal microscope equipped with a 40×/NA 1.3 oil objective lens.

### Light sheet-based fluorescence microscopy

Spheroids were mounted onto a pinhole-containing sample holder with a drop of 1% low-melt agarose (Carl Roth). The specimen was inserted into a PBS-filled microscope chamber and z-stack data with a spacing of 2.5 µm were recorded with an Andor Clara camera (Andor) using a 2.5/NA 0.06 illumination and a 10×/NA 0.3 detection objective lens.

### Image analysis

This image analysis pipeline was performed for time lapse images of spheroid formation, microscope controls and endpoint images. To detect the area occupied by the spheroid, an automated pipeline was developed with *Mathematica* (version 10). The following steps were performed: (a) images were imported whereby all following steps were performed in parallel on at least twelve processors for the individual slices, (b) the mean intensity of the background was subtracted from the image, (c) the image was convolved with a Gaussian kernel (r=35-60), resulting in a highly blurred image, (d) foreground and background were separated by binarisation, (e) holes within foreground objects were filled, (f) the number of foreground pixels was determined and converted into the area and (g) data and used parameter values were written in an Excel file (Fig. S1). Additionally, thumbnail images with the object's segmented outline were exported. Stacks of fluorescent images were time-lapse images or endpoint images from different wells.

### Data analysis

Stacks with less than four spheroids leaving the field of view in a row were interpolated by fitting polynomial curves between missing data points. The area occupied by the spheroid at each time point was normalised to the area at time 0 h. For each condition, mean and standard error of the mean at every time point were calculated. The same was done for the control images, which were imaged only at the beginning and the end of the time lapse. Hypothesis testing (Wilcoxon rank sum test with Holm correction) was performed for different time points for each cell line. Different drugs were compared against the DMSO control. Significant difference was calculated at *P*<0.05 (*), *P*<0.01 (**) and *P*<0.001 (***).

The shrinkage of the projected area was linearly approximated by *ΔNA/Δt*, where *ΔNA* is the difference of the normalised areas between two time points and *Δt* is the time interval (here 30 min). For each cell line, mean and standard error of the mean at every time point were calculated. Based on the shrinkage rate of 4T1 cell (these underwent aggregation, compaction and growth), we determined the aggregation phase which was marked by a fast decay of the projected area. The end of this phase was qualitatively approximated at a shrinkage rate of 0.008. We then assumed that the transition between aggregation and compaction occurred when the shrinkage rate dropped below 0.008 for at least 1.5 h. Growth was characterised by a negative shrinkage rate, which dropped below zero for at least 1.5 h.

### Agent-based computational model

To study the cell aggregation dynamics *in silico* in terms of binding activity, we complemented our experimental data with our previously introduced three-dimensional agent-based model (Garg et al., 2015). Simulations were performed with *Mathematica* (version 10). Simulations were performed with 50 cells as previously described (Garg et al., 2015). Several values can be extracted from the simulations at every time point. The normalised area is determined by the area of the convex hull of the cell coordinates projected to the x-y-plane. To determine the optimal parameter values for the model for the different experimental conditions, we fitted the normalised area of the simulations to the normalised area from the experimental data. We first determined the value of the buoyancy parameter for each cell line. We obtained 1 mg/ml for T47D cells, 2 mg/ml for HC11 cells and 4 mg/ml for 4T1 cells. For each buoyancy parameter value, we performed a parameter scan for the binding and unbinding probabilities. We varied the binding probability between 0.05 and 1.0 and the unbinding probability between 0.01 and 0.2. We performed 25 simulations per parameter combination. Out of these 2500 simulations for each cell line, we determined for each condition the 25 simulations that fitted best and calculated the mean and standard deviations of the parameter values for the binding and unbinding parameters. The goodness of fit was obtained by the Akaike information criterion (AIC).

### Software

Image analysis, data analysis and the simulations of the agent-based computational model were performed with *Mathematica* version 10 (Wolfram Research, Inc.).

## Supplementary Material

Supplementary information
