# Supplementary material for: E-cadherin, actin, microtubules and FAK dominate different spheroid formation phases and important elements of tissue integrity
Source: Biol Open. 2018 Dec 21;8(1):bio037051. doi: 10.1242/bio.037051 (PMC6361217; doi:10.1242/bio.037051)
Supplement: Supplementary information [file biolopen-8-037051-s1.pdf]

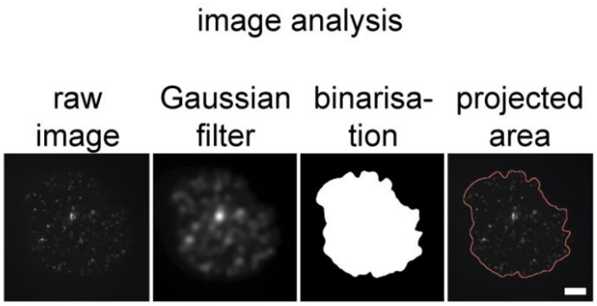

Figure S1. Projected area segmentation of spheroids. Gaussian filter with a large Kernel was applied to fluorescence raw images. The projected areas were measured using filtered binarised images. Images show cell nuclei labelled with eGFP-H2B. The pink outline represents the segmented area. Microscope: Zeiss Cell Observer Z.1, objective: 10x/NA 0.5, scale bar: 100 µm.

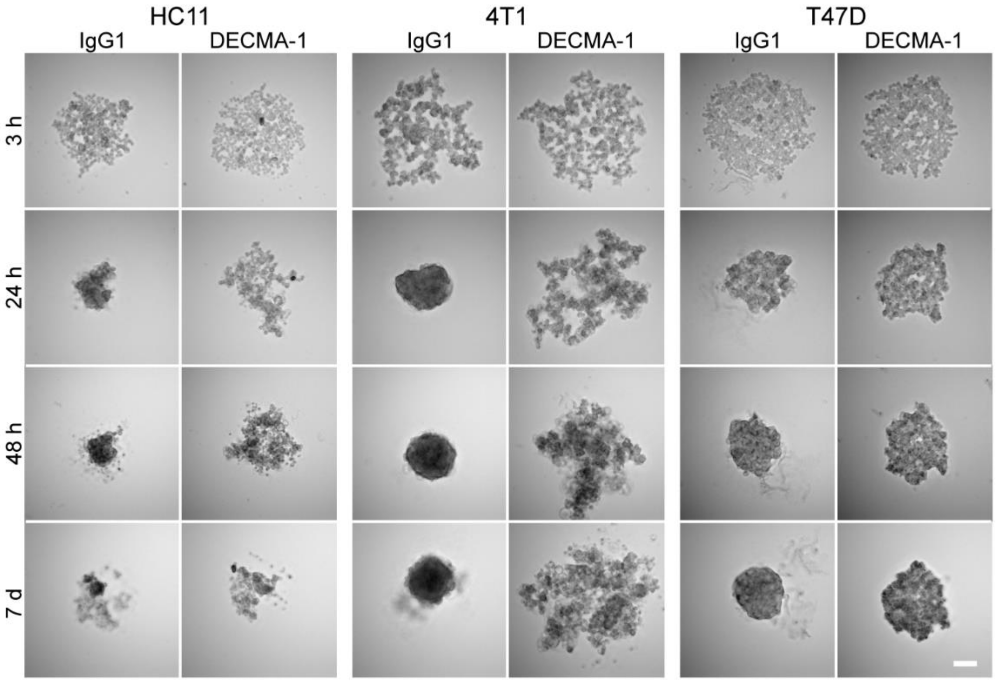

Figure S2. Appearance of spheroids upon block of E-cadherin function. Transmission images show spheroids formation of HC11, 4T1 and T47D cells with or without the block of E-cadherin function using the DECMA-1 antibody. Microscope: Zeiss Cell Observer Z.1, objective: 10x/NA 0.5, scale bar: 100 µm.

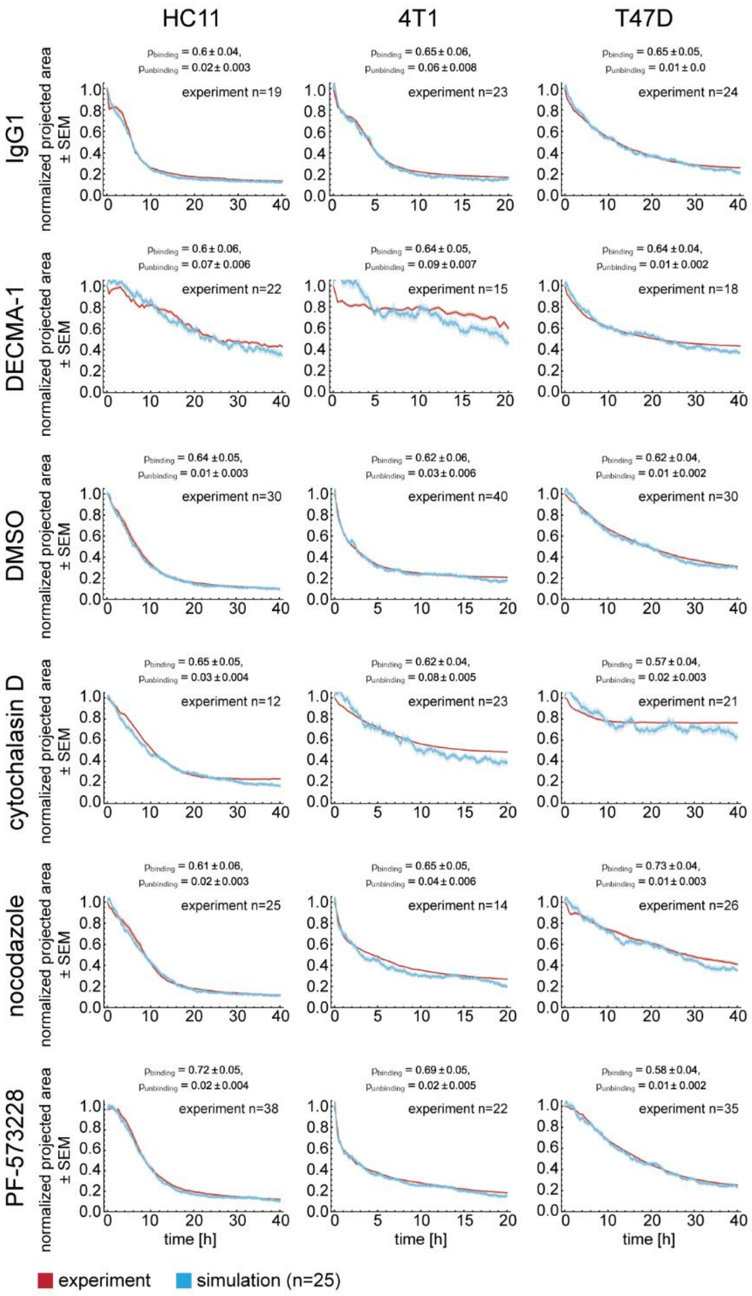

Figure S3. Fitting the computational model to the experimental data and. Plots of the best fits of the agent-based model to the experimental data.

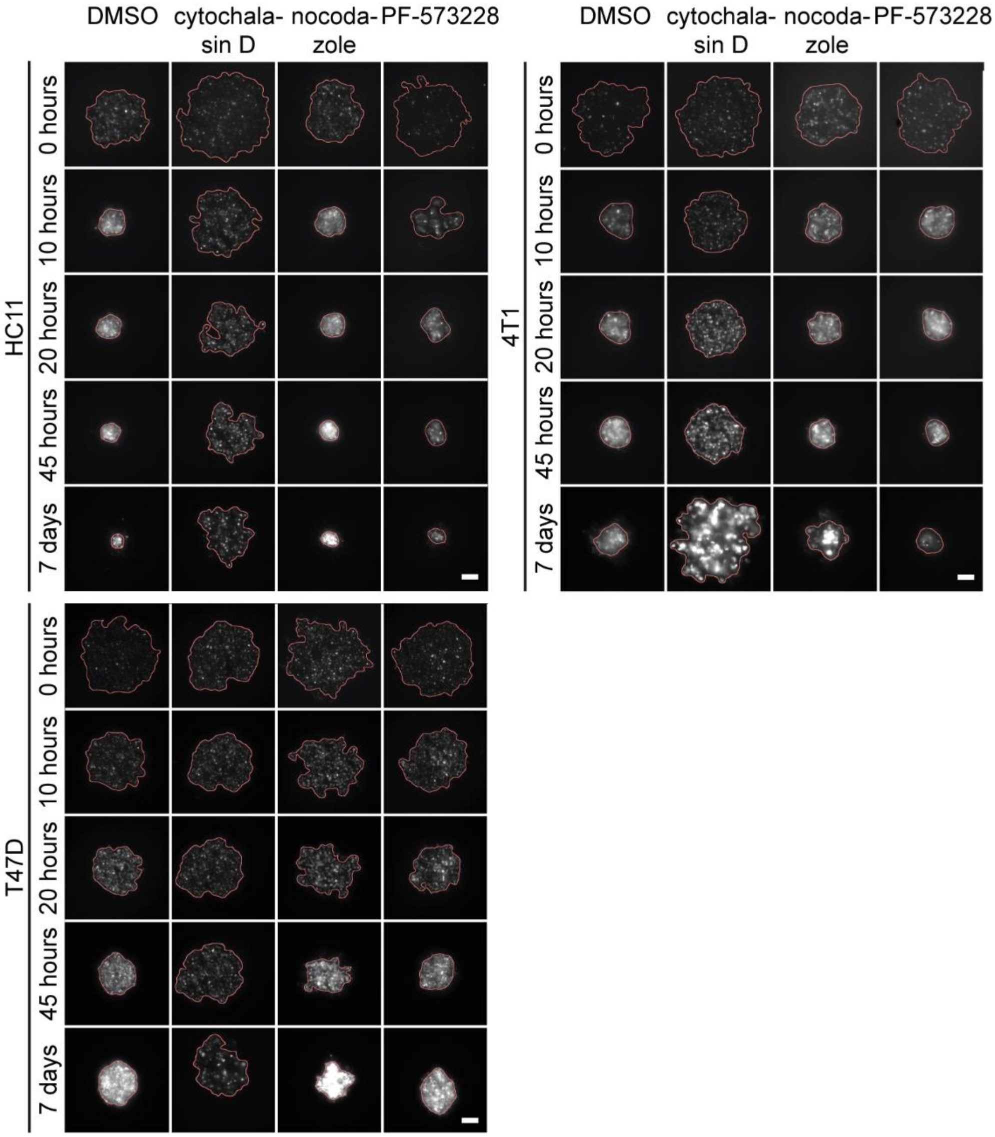

Figure S4. Representative images of the projected area segmentation. Images show spheroid formation for HC11, 4T1 and T47D cells at various time points. Cells express eGFP-H2B to label the cell nuclei. The pink outline represents the segmented area. Microscope: Zeiss Cell Observer Z.1, objective: 10x/NA 0.5, scale bar: 100 µm.

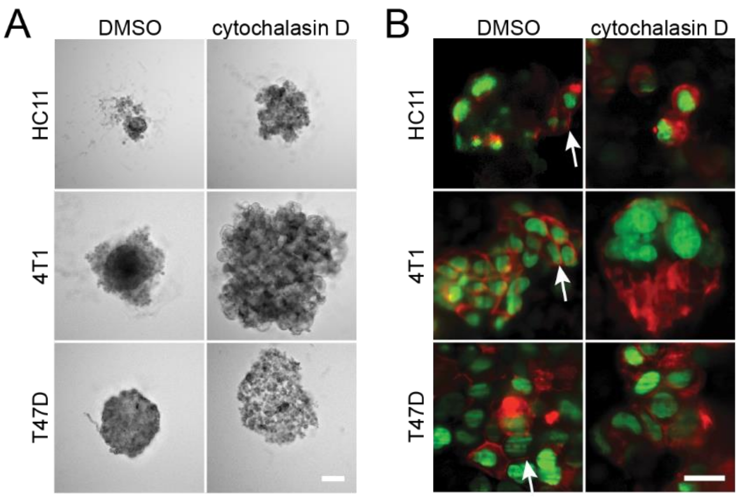

Figure S5. Appearance of spheroids upon disruption of actin filaments. (A) Transmission images show that cells treated with cytochalasin D form lose aggregates after 7 days of spheroid formation. Microscope: Zeiss Cell Observer Z.1, objective: 10x/NA 0.5, scale bar: 100 µm. (B) After 7 days of formation, the actin cytoskeleton (red) is disintegrated in cells from all three cell lines compared to the DMSO control (arrows). 4T1 cell nuclei (green) show massive swelling upon cytochalasin D treatment. The images' background was subtracted and a median filter was applied. Microscope: mDSLM, illumination objective: 2.5x/NA 0.06, detection objective: 20x/NA 0.5, scale bar: 20 µm.

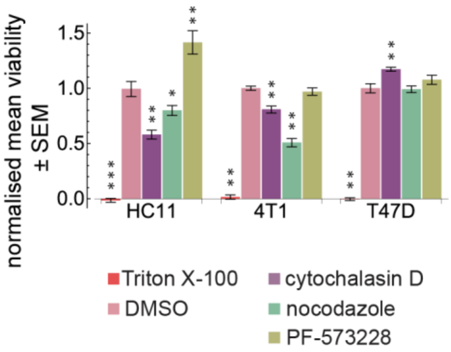

Figure S6. The influence of the drugs on cell death during spheroid formation and cell viability. A cell viability assay was performed on cell monolayers, treated with the drugs for 24 hours. Hypothesis testing was performed using a Wilcoxon rank sum test with Holm correction for multiple testing. Asterisks indicate significant differences (*<0.05, **<0.01, ***<0.001). Drugs were compared against DMSO. Number of independent experiments for HC11, 4T1 and T47D are summarised in Supplementary Table S2.

Biology Open • Supplementary information

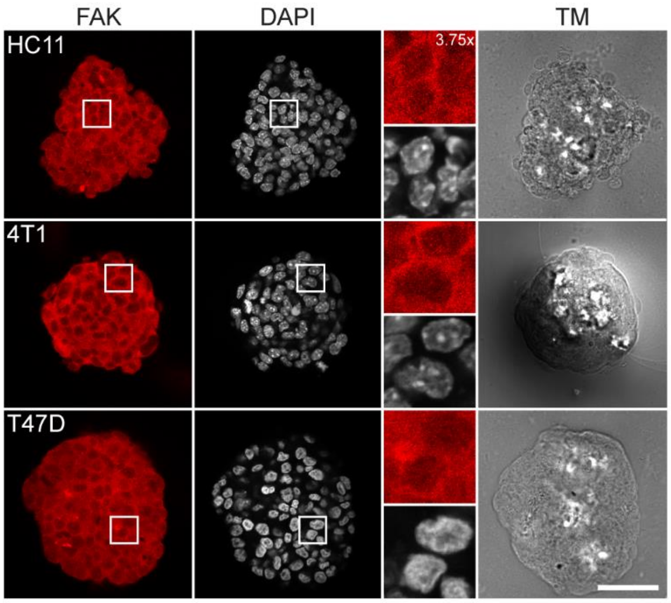

Figure S7. The distribution of FAK in the cytoplasm of cells during spheroid formation. Immunostaining against FAK shows a cytoplasmic distribution in HC11, 4T1 and T47D cell aggregates 48 hours after the initiation of spheroid formation. The staining is excluded from the cell nuclei. DAPI was used to counterstain cell nuclei. A section from the central region of the spheroids is shown. Microscope: Zeiss LSM780, objective: 40x/NA 1.3 oil, scale bar: 50 µm. TM: transmission.

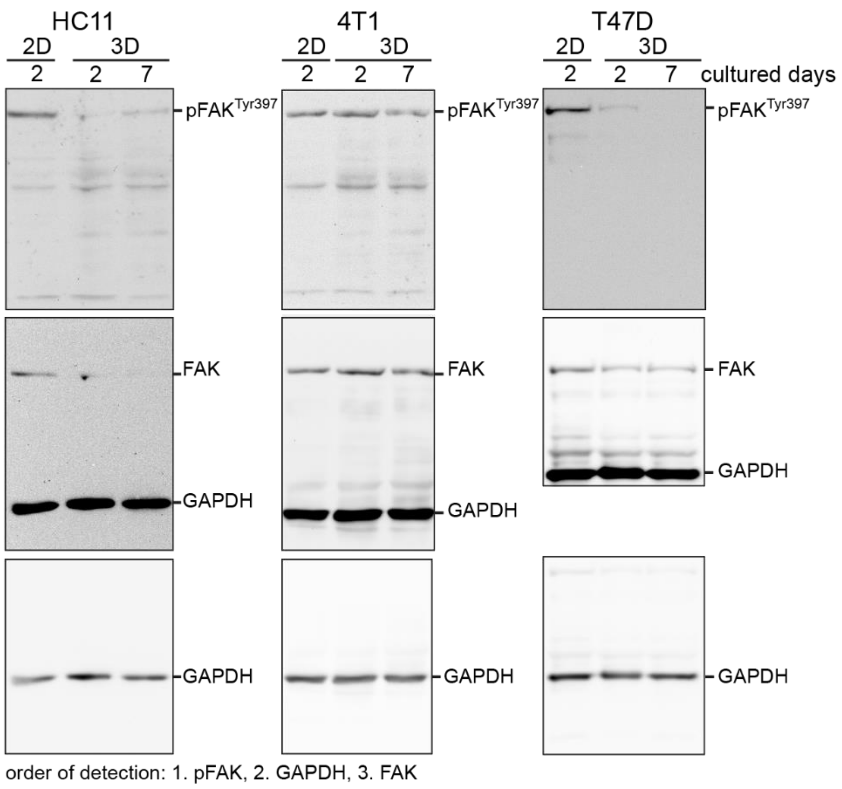

Figure S8. Uncropped Western Blot material as shown in Figure 5.

Biology Open • Supplementary information

Table S1. Light exposure during time lapse does not compromise the formation process. The second column (control TL) shows the normalised projected area after the time lapse experiment of spheroids that have been illuminated every 30 minutes for 48 hours. Last column (control MC) shows the normalised projected area of spheroids, which were not continuously exposed to light, but only once at the beginning and the end of the experiment. TL: time lapse, MC: microscope control.

**HC11**	**Control TL ± SEM**	**Control MC ± SEM**
DMSO (0.5%)	0.09 ± 0.01	0.12 ± 0.03
cytochalasin D (2.5µM)	0.25 ± 0.05	0.26 ± 0.06
nocodazole (5µM)	0.10 ± 0.02	0.11 ± 0.02
PF-573228 (1µM)	0.11 ± 0.02	0.12 ± 0.042
IgG1 (10µg/ml)	0.13 ± 0.03	0.15 ± 0.03
DECMA-1 (10µg/ml)	0.41 ± 0.06	0.45 ± 0.09
**4T1**	**Control TL ± SEM**	**Control MC ± SEM**
DMSO (0.5%)	0.23 ± 0.03	0.25 ± 0.04
cytochalasin D (2.5µM)	0.55 ± 0.06	0.56 ± 0.10
nocodazole (5µM)	0.21 ± 0.02	0.21 ± 0.01
PF-573228 (1µM)	0.11 ± 0.01	0.11 ± 0.01
IgG1 (10µg/ml)	0.18 ± 0.02	0.18 ± 0.03
DECMA-1 (10µg/ml)	0.52 ± 0.09	0.49 ± 0.05
**T47D**	**Control TL ± SEM**	**Control MC ± SEM**
DMSO (0.5%)	0.28 ± 0.04	0.26 ± 0.03
cytochalasin D (1µM)	0.77 ± 0.06	0.73 ± 0.05
nocodazole (0.5µM)	0.38 ± 0.08	0.37 ± 0.10
PF-573228 (1µM)	0.23 ± 0.03	0.21 ± 0.02
IgG1 (10µg/ml)	0.25 ± 0.04	0.24 ± 0.03
DECMA-1 (10µg/ml)	0.43 ± 0.03	0.44 ± 0.04

Biology Open • Supplementary information

Table S2. Number of independent experiments.

**Figure 2 (48h TL)**	**HC11**	**4T1**	**T47D**
IgG1	19	23	24
DECMA-1	22	15	18

**Figure 2 (7 days EP)**	**HC11**	**4T1**	**T47D**
IgG1	19	23	24
DECMA-1	20	6	18

**Figure 3 (48h TL)**	**HC11**	**4T1**	**T47D**
DMSO	30	40	30
cytochalasin D	12	23	21
nocodazole	25	14	26
PF-573228	38	22	35

**Figure 3 (7 days EP)**	**HC11**	**4T1**	**T47D**
DMSO	21	31	21
cytochalasin D	12	23	21
nocodazole	21	13	26
PF-573228	29	19	32

**Supplementary Fig. S6**	**HC11**	**4T1**	**T47D**
Triton X-100	12	9	9
DMSO	12	9	9
cytochalasin D	12	9	9
nocodazole	12	9	9
PF-573228	12	9	9

TL: time lapse, MC: microscope control, TP: time point, EP: end point

Biology Open • Supplementary information

Table S3. Western blot quantitative densitometry of protein expression. The value for pFAK^{Tyr397} was is shown relative to the loading control, GAPDH. The values for the respective cell lines were normalised to the monolayer cultures.

	HC11				4T1				T47D		
	**2D**	**3D**			**2D**	**3D**			**2D**	**3D**	
	**2 days**	**2 days**	**7 days**		**2 days**	**2 days**	**7 days**		**2 days**	**2 days**	**7 days**
**HC11-1**	1.00	0.07	0.18	**4T1-1**	1.00	1.13	0.71	**T47D-1**	1.00	0.43	-0.12
**HC11-2**	1.00	0.01	0.08	**4T1-2**	1.00	0.14	1.30	**T47D-2**	1.00	0.20	-0.01
								**T47D-3**	1.00	0.09	0.00
